# *In Vitro* Evaluation of Dietary Fiber Anti-Infectious Properties against Food-Borne Enterotoxigenic *Escherichia coli*

**DOI:** 10.3390/nu13093188

**Published:** 2021-09-14

**Authors:** Thomas Sauvaitre, Claude Durif, Adeline Sivignon, Sandrine Chalancon, Tom Van de Wiele, Lucie Etienne-Mesmin, Stéphanie Blanquet-Diot

**Affiliations:** 1 UMR 454 UCA-INRAE Microbiologie Environnement DIgestif et Santé (MEDIS), Université Clermont Auvergne, 63000 Clermont-Ferrand, France; Thomas.SAUVAITRE@uca.fr (T.S.); Claude.DURIF@uca.fr (C.D.); sandrine.chalancon@uca.fr (S.C.); lucie.etienne-mesmin@uca.fr (L.E.-M.); 2 Faculty of Bioscience Engineering Center for Microbial Ecology and Technology (CMET), Ghent University, 9000 Ghent, Belgium; Tom.VandeWiele@UGent.be; 3 UMR 1071 UCA Inserm USC-INRAE 2018 Microbes Intestin Inflammation et Susceptibilité de l’Hôte (M2iSH), Université Clermont Auvergne, 63000 Clermont-Ferrand, France; Adeline.SIVIGNON@uca.fr

**Keywords:** dietary fibers, food-borne pathogen, Enterotoxigenic *Escherichia coli*, virulence, adhesion, intestinal cells, LT toxin, mucus

## Abstract

Dietary fibers have well-known beneficial effects on human health, but their anti-infectious properties against human enteric pathogens have been poorly investigated. *Enterotoxigenic Escherichia coli* (ETEC) is the main agent of travelers’ diarrhea, against which targeted preventive strategies are currently lacking. ETEC pathogenesis relies on multiple virulence factors allowing interactions with the intestinal mucosal layer and toxins triggering the onset of diarrheal symptoms. Here, we used complementary *in vitro* assays to study the antagonistic properties of eight fiber-containing products from cereals, legumes or microbes against the prototypical human ETEC strain H10407. Inhibitory effects of these products on the pathogen were tested through growth, toxin production and mucus/cell adhesion inhibition assays. None of the tested compounds inhibited ETEC strain H10407 growth, while lentil extract was able to decrease heat labile toxin (LT) concentration in culture media. Lentil extract and specific yeast cell walls also interfered with ETEC strain H10407 adhesion to mucin beads and human intestinal cells. These results constitute a first step in the use of dietary fibers as a nutritional strategy to prevent ETEC infection. Further work will be dedicated to the study of fiber/ETEC interactions within a complex gut microbial background.

## 1. Introduction

Dietary fibers are carbohydrate polymers with 10 or more monomeric units, which are not hydrolyzed by endogenous enzymes in the human small intestine, thus providing preferential substrates for gut microbes [1]. Most of the dietary fibers consumed by humans are of plant origin, but some of them are also derived from animals, fungi or bacteria [2]. They have a well-known beneficial health effect in humans, such as transit regulation, slowing down of glucose absorption, immune system modulation and support of gut microbiota diversity [3,4]. Insoluble dietary fiber particles have even been recently shown to constitute a microbiota niche on their own [5,6]. Another understudied effect of dietary fibers is their ability to prevent enteric infections [7]. Scarce *in vitro* studies have already shown the antagonistic properties of fibers against various enteric bacterial pathogens, mostly through a direct bacteriostatic effect, anti-adhesion properties on intestinal cells [8,9,10,11] or a decoy for pathogen/toxin binding to mucosal polysaccharides [12,13,14,15]. Interestingly, dietary fibers could also lure the resident gut microbiota from mucus consumption, thereby impeding access to the underlying epithelium to pathogen such as *Citrobacter rodentium* [16]. Therefore, dietary fibers may be considered a promising alternative strategy when available therapy for the management of enteric infection is limited, as met with Enterotoxigenic *Escherichia coli* (ETEC) [17]. ETEC is the main agent of traveler’s diarrhea, responsible for hundreds of millions of diarrheal episodes worldwide [18,19]. ETEC has a preferential tropism for the distal part of the small intestine [20,21], where bacteria have to degrade mucus and adhere to the intestinal epithelium using mucinases and a myriad of adhesins [22,23,24]. Then, the hallmark of ETEC infection is the production of two toxins, the heat-labile (LT) and heat-stable (ST) enterotoxins, which through binding to their respective receptors are both leading to hypersecretion of H_2_O and Cl^−^ at the root of watery cholera-like diarrhea [25,26]. To date, very few studies have addressed dietary-fiber effects upon ETEC strains from human origin. Only milk oligosaccharides and plantain soluble fibers were proven to reduce ETEC adhesion to Caco-2 intestinal epithelial cells [27,28,29]. Here, we investigated using complementary *in vitro* approaches the potential antagonistic properties of eight fiber-containing products against the prototypical human ETEC strain H10407. We assessed the effect of dietary-fiber-containing products on bacterial growth and LT toxin production in broth media, as well as their anti-adhesive properties on mucins and human intestinal epithelial cells.

## 2. Materials and Methods

### 2.1. Dietary-Fiber-Containing Products

The main characteristics of the eight dietary-fiber-containing products tested in this study are summarized in Table 1. Among them, some were kindly provided by local companies, while others were purchased from a supplier (Merck, Darmstadt, Germany) or extracted in the laboratory from raw products. Before this extraction, lentils, red beans and oats were prepared according to their usual households of consumption. Briefly, red beans and oat flakes were soaked in water overnight and 10 min, respectively. Red beans and lentils were separately boiled (30 min). Then, all products were washed in sterile distilled water, ground at maximum speed in a blender (8010S, Waring, Torrington, Connecticut, USA) until homogeneity and filtered through a 0.9 mm diameter pore filter. Per 200 g of raw products, 10 g of pancreatin (P1750, Merck, Darmstadt, Germany) was added to 200 mL of sterile distilled water and centrifuged (8000× *g*, 30 min, 4 °C). The supernatant was collected and added to the ground material with 3.2 mg trypsin (T0303, Merck, Darmstadt, Germany). Digestion was performed for a total duration of 6 h (100 rpm, 37 °C). To precipitate soluble fibers, 3 volumes of 96% ethanol were then added to the mixture under agitation (4 °C, 100 rpm, 1 h). The solution was centrifuged (2500× *g*, 15 min, 4 °C) and the pellet was washed 3 times in 75% ethanol. Finally, the pellet was dried in an incubator (overnight, 42 °C) and then finely ground at full speed under sterile conditions in a blender (8010S, Waring, Torrington, Connecticut, USA). Fiber content of the eight products was analyzed by an external company (CAPINOV, Landerneau, France) according to the AOAC 985.29 method [30], except for wheat starch (resistant starch content was directly indicated by the provider). If the products were not sterile as determined by plating on plate counting agar, they were autoclaved (121 °C, 15 min). In all *in vitro* experiments, products were used at the final fiber concentration of 2 g·L^−1^.

### 2.2. ETEC Strain and Growth Conditions

The prototypical ETEC strain H10407 serotype O78:H11:K80 (ATCC^®^ 35401, LT+, ST+, CFA/I+) isolated in Bangladesh from a patient with a cholera-like syndrome [31] was used in this study. Bacteria were routinely grown under agitation (125 rpm, overnight, 37 °C) in Luria Bertani (LB) broth until OD 600 nm = 0.6 (stationary phase).

### 2.3. Growth Assay

ETEC strain H10407 (10^6^ CFU·mL^−1^) was allowed to grow aerobically for 6 h at 37 °C under 100 rpm agitation, in complete LB (Sigma, St. Louis, MO, USA) or minimal M9 medium, with or without each fiber-containing product. Medium was regularly sampled and plated onto LB agar for ETEC numeration. Three independent biological replicates were performed.

### 2.4. LT Toxin Overnight Production

LT production was assayed by cultivating ETEC strain H10407 with or without fiber-containing products in overnight Casamino Acids-Yeast Extract (CAYE) medium at 37 °C under agitation (100 rpm) [32]. After overnight culture, medium was centrifuged (3000× *g*, 5 min, 4 °C,) and toxin concentrations were measured in the supernatant by ELISA assay as previously described [33,34]. Optical density was read at 450 nm using an EPOCH multiplate spectrophotometer (BIOTEK, Winooski, Vermont, USA). Three independent biological replicates were performed.

### 2.5. Mucin Beads

Mucin beads were obtained as already described [35]. Mucin from porcine stomach type III (Sigma-Aldrich, Saint-Louis, MO, USA) was diluted in sterile distilled water, at a concentration of 5% (*w/v*). Sodium alginate (Sigma-Aldrich, Saint-Louis, MO, USA) was added at a concentration of 2% (*w/v*). To produce mucin alginate beads, the mixture was dropped using a peristaltic pump into a 0.2 M solution of sterile CaCl_2_ under agitation (100 rpm). Beads (diameter: 4.5 mm in average) were then kept at 4 °C (no more than 24 h prior use).

### 2.6. Mucin Bead Adhesion Assay

Adhesion assays were carried out with mucin beads resuspended in 50 mL PBS pH 6.8 with or without fiber-containing products. ETEC strain H10407 was added at 10^7^ or 10^8^ CFU·mL^−1^ for a 30 min or 1 h contact period, respectively. Mucin-beads were then washed three times with 40 mL sterile physiological water and then crushed in 19.8 mL physiological water with an ultra turrax apparatus until homogeneity (IKA, Staufen, Germany). Adhered bacteria were numerated by plating onto LB agar. Each experiment was repeated at least four times.

### 2.7. Caco-2/HT29-MTX Cell Cultures

Caco-2 and HT29-MTX cells were maintained in Dulbecco’s Modified Eagle Medium (DMEM, Gibco, Life Technologies, Paisley, UK) containing glucose and glutamine, supplemented with non-essential amino acid (Gibco, Life Technologies, Paisley, UK) and antibiotic-antimycotic solution (Gibco, Life Technologies, Paisley, UK). Media were also supplemented with 20 and 10% Foetal Bovine Serum (FBS, Thermo Fisher Scientific, Waltham, Massachusetts, USA) for Caco-2 and HT29-MTX cells, respectively. For experimental studies, Caco-2 and HT29-MTX cells were seeded at a density of 10^5^ cells/well on 12 wells plates (Thermo Fisher Scientific, Waltham, Massachusetts, USA) at a ratio 70:30. The co-culture was allowed to differentiate for 18 days in medium with 20% heat-inactivated FBS in an atmosphere of 5% CO_2_ at 37 °C. The growth medium was replaced every 2 days.

### 2.8. Adhesion Tests on Caco-2/HT29-MTX Co-Culture Model

Cells were pre-treated or not with fiber-containing products for a 3-h period. Cells were then infected with ETEC strain H10407 at a multiplicity of infection (MOI) of 100 for 3 h in antibiotic–antimycotic free medium. After three washes with PBS pH 7.2 at 4 °C (Thermo Fisher Scientific, Waltham, Massachusetts, USA), cells were lysed with 1 mL of 1% Triton X-100 (Sigma, St. Louis, MO, USA). Serial dilutions of lysed cells were plated onto LB agar to determine the number of adhered bacteria. Each experiment was repeated at least six times.

## 3. Results

### 3.1. All Fiber-Containing Products Have No Effect on ETEC Growth in Complete Nutritive Medium

**Figure 1:**
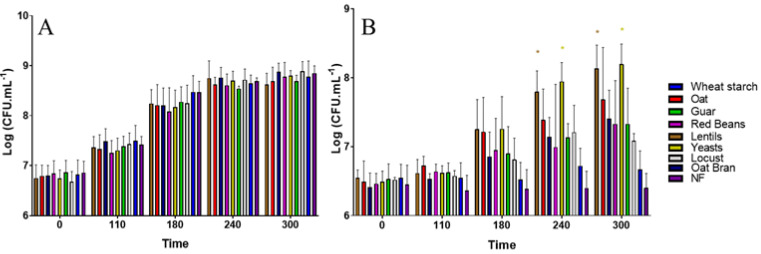
Effect of fiber-containing products on the time course of ETEC strain H10407 bacterial growth in complete LB medium (**A**) or in M9 minimal medium (**B**). Fiber-containing products were tested at 2 g·L^−1^ of final fiber content. Each bar represents the mean of three biological independent replicates (±SD). Results are expressed as mean log10 CFU·mL^−1^. Significance with the control condition was determined by Dunnett’s multiple comparisons test (*: *p* < 0.05). NF = control condition with no product added, SD = standard deviation.

When ETEC bacteria were grown in LB-rich medium (Figure 1A), no statistical difference was observed between each fiber-supplemented condition and the negative control (no fiber) according to Dunnett’s multiple comparisons test. The growth curves were similar whatever the conditions tested, with all culture reaching between 6.10^8^ and 8.10^8^ CFU·mL^−1^ at the end of the experiment. Therefore, none of the eight screened products was able to reduce ETEC growth in complete culture medium. In M9 minimal medium, all of the fiber-containing products showed a tendency to sustain ETEC growth compared to the non-treated condition, with a clear product effect (Figure 1B). In particular, lentils and the specific yeast cell walls from *Saccharomyces cerevisiae* AQP 12,260 led to more than 1-log difference with the control condition after 5 h. These differences became statistically different at 240 and 300 min according to Dunnett’s multiple comparisons test (*p* < 0.05).

### 3.2. Lentil Fibers Decreases LT Toxin Production

**Figure 2:**
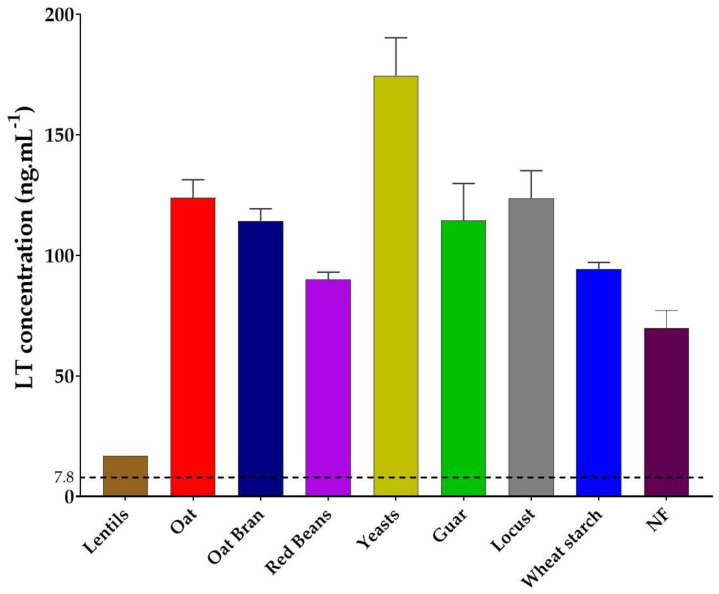
Effect of fiber-containing products on LT toxin concentration after an overnight culture of ETEC strain H10407 in CAYE medium. Fiber-containing products were tested at 2 g·L^−1^ of final fiber content. Results are expressed as mean ng·mL^−1^ of toxin (±SD) of three independent biological replicates. For lentil extracts, only one biological replicate is represented as toxin was not detected in the two other replicates. No statistical test was performed because of these two under-threshold results. The detection threshold (7.8 ng·mL^−1^) is indicated by a dotted black line. NF = control condition with no product added, SD = standard deviation.

We also evaluated the direct effect of the eight fiber-containing products on LT toxin concentration in CAYE medium, known to support toxin production [30]. In the absence of fiber-containing product (control condition), ETEC strain H10407 produced on average 69.7 ± 7.4 ng·mL^−1^ of LT toxin (Figure 2). With most of the tested products, a higher LT toxin concentration was measured (from 90.0 ± 2.9 ng·mL^−1^ for red beans to 174.5 ± 15.7 ng·mL^−1^ for specific yeast cell walls). In sharp contrast, the lentil-derived fibers widely reduced LT toxin concentrations. One biological replicate showed a concentration of 16 ng·mL^−1^ of toxin, much lower than the non-treated condition (NF) and the toxin was not detected in the two other replicates (levels below the detection threshold). When incubated with pure LT toxin (500 ng·L^−1^), lentil extracts (from 0 to 8 g·L^−1^ of fiber content) significantly decreased toxin amount at the highest dose (*p* < 0.05) but had no effect at the 2 g·L^−1^ concentration used throughout this study (data not shown).

### 3.3. Specific Yeast Cell Walls and Lentil Fibers Inhibit ETEC Adhesion to Mucin Beads

**Figure 3:**
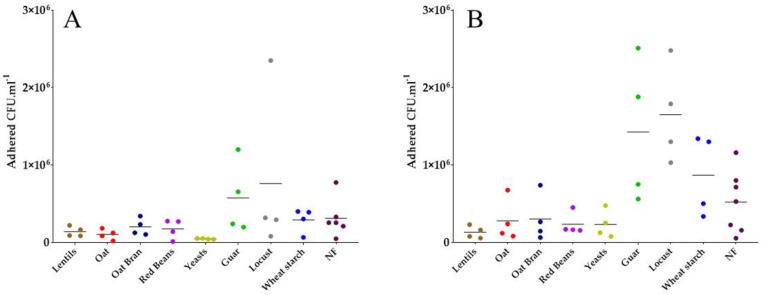
Effect of fiber-containing products on ETEC adhesion on mucin beads, when inoculating with 10^7^ (**A**) or 10^8^ (**B**) CFU·mL^−1^, during 30 min (**A**) or 1 h (**B**) contact period. Fiber-containing products were tested at 2 g·L^−1^ of final fiber content. Each biological replicate and its mean is represented (*n* = 4–7). Results are expressed as adhered CFU·mL^−1^ of crushed bead solution. No significance with the control condition was found according to Tukey’s multiple comparison tests. NF = control condition with no product added.

Next, a mucin-bead adhesion assay aimed at investigating whether some fiber-containing products were able to reduce pathogen adhesion to intestinal mucus. Different experimental conditions were tested: initial bacterial concentration of 10^7^ CFU·mL^−1^ and a 30 min contact time in the first experiment (Figure 3A), parameters that were both increased in the second assay with 10^8^ CFU·mL^−1^ and a 60 min contact time (Figure 3B). Both experiments showed a clear tendency of lentils, oat, oat bran, red beans and specific yeast cell walls containing fiber products to reduce ETEC adhesion to mucin beads (Figure 3B). In particular in the first assay (Figure 3A), specific yeast cell walls reduced ETEC adhesion more than 6-fold. In the second assay (Figure 3B), lentils reduced ETEC adhesion of more than 4-fold.

### 3.4. Most Fiber-Containing Products Inhibit ETEC Adhesion to Caco-2/HT29-MTX Co-Culture

**Figure 4:**
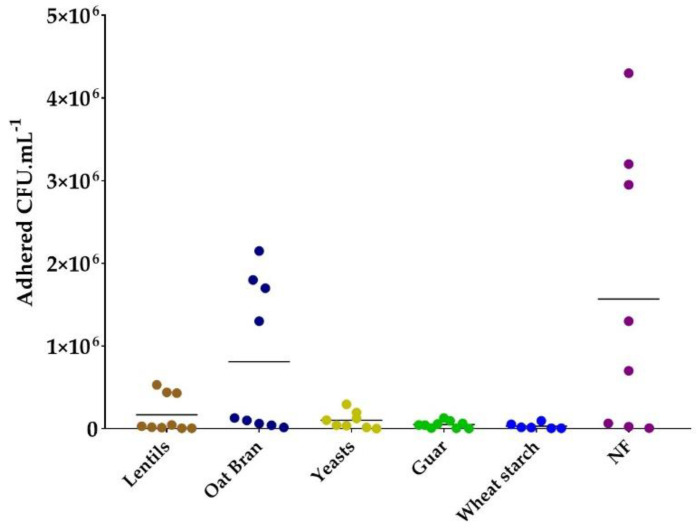
Modulation of ETEC adhesion to co-culture of Caco-2/HT29-MTX cells by fiber-containing products. Fiber-containing products were tested at 2 g·L^−1^ of final fiber content. Each biological replicate and their mean are represented (*n* = 6–9). Results are expressed as adhered CFU·mL^−1^ of cellular lysate. No significance with the control condition was found according to Tukey’s multiple comparison tests. NF = control condition with no product added.

To further address the potential of fiber-containing products to modulate ETEC adhesion to the human intestinal epithelium, we performed adhesion experiments on a co-culture model of Caco-2/HT29-MTX cells, respectively differentiating in enterocytes and mucus-secreting goblet cells. These experiments were performed with a lower number of products, selected from the results of previous *in vitro* experimentations. All tested products except oat bran showed a trend in reducing ETEC adhesion levels (Figure 4). Average adhesion levels were 50-, 30- and 15-fold lower compared to the control condition (no fiber-containing product added), for wheat starch, guar and specific yeast cell walls, respectively.

## 4. Discussion

The current study aimed at evaluating the antagonistic properties of a broad range of dietary-fiber-containing products against the prototypical human ETEC strain H10407 using various complementary *in vitro* assays. Fibers from lentils and specific yeast cell walls showed the most interesting inhibitory properties because of their LT toxin lowering and mucosal adhesion inhibiting properties, respectively. The products tested herein have different origins (vegetal or microbes) and thus contain different types of soluble and/or insoluble fibers, among which are resistant starch for wheat starch, beta-glucans for oats and specific yeast cell walls, mannans for specific yeast cell walls, galactomannan for guar and locust bean gums, celluloses and hemicelluloses for lentils [36]. All the products were tested at the physiological dose of 2 g of fibers per liter, considering both the ingested amount in a Westernized diet that ranges from 10 to 30 g per day [37,38,39] and the dilution by digestive fluids of nearly 10 L per day [40]. This amount of 2 g·L^−1^ is in the range of tested fiber concentrations against intestinal pathogens *in vitro* (up to 10 g·L^−1^ for complex polysaccharides in cellular assays), as recently reviewed [5]. The ETEC strain was also used in the *in vitro* assays at a physiological concentration as infectious dose in humans varies from 10^5^ to 10^10^ of ingested bacteria [41,42,43,44].

The beneficial effects of dietary fibers on human health is now well acknowledged, but their ability to exert antagonistic effects against enteric pathogens remain poorly studied [7,36,45,46,47]. To date, the vast majority of studies investigating the potential of fibers in the fight against ETEC-associated infections have been performed on porcine ETEC strains [48,49,50,51,52], while studies involving ETEC strains from human origins are scarce [28,29,53].

Fibers can act at different levels of the ETEC pathological process. A first target in our study was the observed reduction in the number of bacteria able to reach the pathogen’s site of action in the distal part of the human small intestine [21]. Then, we first investigated the direct antagonist effect of fiber-containing products on ETEC strain H10407 growth in classical culture media. None of the tested products was able to reduce pathogen growth in LB complete medium. This is not surprising since to our knowledge only chitosan, a human-engineered fiber, was previously shown to inhibit pathogen growth among enterohemorrhagic *E. coli* (EHEC) [8]. Moreover, tested products were all sustaining ETEC growth when using M9 minimal medium, most probably due to the presence of non-fiber components in the fiber-containing products, as *E. coli* strains are not known to be able to degrade complex polysaccharides [54]. When translated to the complex nutritional background of the human gut, this is certainly not an issue since ETEC will encounter many other nutrient sources than the ones provided by our fiber-containing products.

In a second step, since toxin production is a key feature in ETEC physiopathology, we assessed the effect of fiber-containing products on LT toxin production. To our knowledge, only one study has previously reported an indirect effect of dietary fibers on ETEC toxin. Indeed, short chain fatty acid (SCFAs), that are major end products of dietary fiber metabolism by gut microbiota, significantly reduced or even abolished LT toxin production at a concentration of 2 g·L^−1^ in CAYE culture medium [53]. In our study, lentil extract induced a decrease in LT enterotoxin concentration in the supernatant of overnight ETEC strain H10407 culture in CAYE medium. Several hypotheses have been raised related to the mechanisms of action. First, lentil extract may repress ETEC LT toxin production at the transcriptional or translational levels, but there are no data in the literature to support such a hypothesis. Then, if the LT toxin production is not impacted by lentil extract, we can imagine that the inhibition can occur at the detection step. To challenge this hypothesis, we evaluated the effect of lentil extracts on pure LT toxin solutions and showed that the inhibitory effect was partially conserved but not significant at the dose of 2 g·L^−1^ of fiber used in this study. Such inhibition can occur at different steps of the ELISA assay, but we can speculate that the toxin binds to some lentil components that act as decoys, preventing interactions with antibodies. In a next step, we could investigate the inhibitory effect of fiber-containing products on ETEC toxin production *via* gut microbiota modulation.

Lastly, fibers can also favor the exclusion of pathogens from mucosal surface by presenting potential binding sites and thus acting as decoys. Many dietary fibers originating from milk, plants and microorganisms have already proven efficiency in reducing adhesion to mucus [51,55], erythrocytes [49,56], Caco-2 cell line [48,57] or cells from porcine jejunal epithelium [50,52,58] for ETEC strains from animal origins. Here, we investigated the ability of some of our fiber-containing products to distract the human ETEC strain H10407 from mucosal-like surfaces. First, by using a mucin-bead adhesion assay we demonstrated that lentil extracts and yeast cell walls could decoy the ETEC strain H10407 from mucus polysaccharides adhesion. Compared to other mucus.-integrating models used to test inhibitory properties of fibers [51,55], the use of mucin beads under constant agitation eliminates the possibility of non-specific bacterial exclusion by fiber sedimentation on the mucus compartment. Second, to integrate the host part, we used cellular-adhesion experiments with the Caco-2/HT29-MTX cellular co-culture model. This model includes Caco-2 enterocytes-like cells and HT29-MTX cells secreting mucus polysaccharides [59,60,61]. To date, only milk oligosaccharides [27,29] and soluble plantain fibers at a dose of 5 g·L^−1^ [28] have shown efficiency to reduce adhesion of human ETEC strains other than H10407 to the Caco-2 cell line. Our experiments showed a global trend towards an inhibitory effect of yeast-specific cell walls and lentils, but also of other fiber-containing products such as guar gum and wheat starch. As previously observed, such effects can be due to a decoy effect from mucus adhesion. Moreover, it is well known that ETEC adhesins are able to recognize specific receptors on cell surfaces, such as glycosphingolipids [62,63,64], mannosylated proteins [65] and fibronectin [66]. This implies that some components found in all tested compounds, probably fibers, may be able to bind to such cellular receptors, thereby blocking bacterial attachment. Lastly, we checked using Trypan blue exclusion assay that specific yeast walls and lentil extract had no effect on ETEC-induced cytotoxicity in intestinal Caco-2 and HT-29-MTX cells (data not shown).

## 5. Conclusions

Taken together, our results suggest that among the tested fiber-containing products, lentils and yeast-specific cell walls could present promising anti-infectious activities against the human reference ETEC strain H10407. These effects seem to be mediated through a multi-targeted pathway, namely inhibition of toxin production and reduction of adhesion to mucins and intestinal epithelial cells. The associated mechanism of action obviously needs to be further investigated. Next steps will be dedicated to the testing of selected fiber-containing products in more complex experimental set-ups reproducing the physiological conditions of the human digestive environment in a more representative manner and including gut microbiota, which is a key player in gut homeostasis and fiber degradation. This study is the first step in the use of dietary fibers as a new nutritional strategy to prevent ETEC-induced traveler’s diarrhea.

## Figures and Tables

**Table 1:** Characteristics of dietary-fiber-containing products. The product origin and source, their solubility at 2 g·L^−1^ in water, their fiber content, and the analysis method used for determining fiber content are indicated in the table.

Extract/Product	Origin	Product Source	Solubility at 2 g·L^−1^ in Water	Fiber Content (g·100 g^−1^)	Analysis Method
Green lentils	Plants	Home made	Insoluble	41.4	AOAC 985.29
Guar gum	Plants	Commercially available	Soluble	84.3	AOAC 985.29
Locus bean gum	Plants	Commercially available	Soluble	83.3	AOAC 985.29
Oat	Plants	Home made	Insoluble	19.8	AOAC 985.29
Oat bran	Plants	Provided by local companies	Insoluble	44.4	AOAC 985.29
Red beans	Plants	Home made	Insoluble	53	AOAC 985.29
Wheat starch	Plants	Provided by local companies	Soluble	17	Resistant starch content communicated by provider
Specific yeast cell walls (from *Saccharomyces cerevisiae*)	Microorganisms	Provided by local companies	Insoluble	62.6	AOAC 985.29

## Data Availability

Not applicable.
